# Antimicrobial Properties of Newer Calcium Silicate-Based Pulp-Capping Agents Against Enterococcus Faecalis and Streptococcus Mutans: An In-Vitro Evaluation

**DOI:** 10.7759/cureus.70459

**Published:** 2024-09-29

**Authors:** Niranjana M, Sunil Jose, George Thomas, Arun Shyam, Aparna M

**Affiliations:** 1 Conservative Dentistry and Endodontics, Mahe Institute of Dental Sciences & Hospital, Mahe, IND; 2 Conservative Dentistry and Endodontics, Kannur Dental College, Anjarakandy, IND

**Keywords:** agar diffusion test, antibacterial effects, bacteria, bioactive materials, pulp capping agents, zone of inhibition

## Abstract

Aim: This study aimed to evaluate the antibacterial activity of calcium silicate-based pulp-capping agents against *Enterococcus faecalis* and *Streptococcus mutans* using an agar diffusion test.

Methods and materials: The agar diffusion method was used to evaluate the antibacterial properties of pulp-capping agents. The materials used included Bio-C® Temp (Angelus, Brazil), Dia-Root™ Bio mineral trioxide aggregate (MTA) (Diadent Europe B.V., Almere, Netherlands), Biodentine™ (Septodont, Saint-Maur-des-Fossés, France), and TheraCal LC (Bisco Inc., Schaumburg, IL). Eighteen petri dishes, nine for *S. mutans* and nine for *E. faecalis*, were divided into four parts each (one for each agent), for a total sample size of 72. The bacterial suspensions were transferred to the petri dishes using a sterile swab. Four wells with a diameter of 4 mm were then punched in each petri dish. The wells were filled with the pulp-capping agents, which had been mixed according to the manufacturer’s instructions, and the petri dishes were incubated. The zone of inhibition was measured at 24 and 48 hours to assess the pulp-capping agents’ antimicrobial efficacy against *E. faecalis* and *S. mutans*. The readings were tabulated and subjected to statistical analysis.

Results: At 24 hours, the highest zone of inhibition was found in the Biodentine™ group (15.83 ± 0.79 mm), followed by Dia-Root™ Bio MTA (14.5 ± 0.88 mm), TheraCal LC® (12.56 ± 0.53 mm), and the shortest in the Bio-C Temp (9.61 ± 0.70 mm) against* S. mutans*. Analysis of variance (ANOVA) test showed a high statistical significance. After 48 hours, there was no statistically significant difference in the mean zone of inhibition.

At 24 hours, the highest zone of inhibition was found in the Biodentine™ group (20.56 ± 0.73 mm), followed by Dia-Root™ Bio MTA (20.06 ± 1.33 mm), TheraCal LC® (18.22 ± 0.97 mm), and the shortest in the Bio-C Temp (14.11 ± 0.78 mm) against* E. faecalis.* The ANOVA test indicated no statistically significant difference between the Biodentine™ and the Dia-Root™ Bio MTA groups. After 48 hours, there was no statistically significant difference in the mean zone of inhibition.

Conclusions: Biodentine™ has higher antibacterial efficacy against *S. mutans*, while Biodentine™ and Dia-Root™ Bio MTA have comparably high antibacterial activity against *S. mutans* and *E. faecalis *at 24 and 48 hours.

## Introduction

Dental caries is one of the oldest documented diseases of the human body. The primary reason for this predilection is the transformation in dietary habits from eating raw food to cooked and processed foods that have a high sugar content. A sugar-rich diet allows microorganisms in the oral cavity to multiply and overwhelm the host defense by producing acids that lead to the breakdown of calcified structures. Once tooth enamel is demineralized, the caries progress rapidly through dentine and reach the vicinity of the pulp. The microbes and their toxins elicit an inflammatory response in the pulp, leading to transient pulpal damage.

Upon identification of caries, the treatment of choice is eradication using mechanical and therapeutic methods before the pulp is further affected. However, such treatment options can fail when the residual microorganisms in dentine are not completely eradicated, which can lead to secondary caries and further pulpal damage. It is important to preserve the vitality of the pulp, as vital pulp tissue is capable of mounting defenses against microbial invasions. Thus, to maintain tooth vitality, pulp capping with appropriate agents below the restoration is important.

Pulp capping can maintain healthy pulp tissue even when the calcified tissues have been compromised by caries, trauma, or restorative procedures. A biocompatible material is placed directly over the pulp or the remaining dentin after the removal of caries. This material must possess direct antibacterial properties to be classified as an ideal material of choice [1].

The commonly used pulp-capping agents include traditional materials, such as calcium hydroxide, and newer materials, such as mineral trioxide aggregate (MTA). Calcium hydroxide was once considered the gold standard for pulp capping, but its compromised adhesion to dentine and tunnel defects in the calcific barrier has led to the introduction of newer materials [2,3].

Calcium silicate-based materials have bioactive properties that allow them to form apatite by utilizing calcium silicates or calcium aluminates [4]. There was a paradigm shift in the treatment outcomes of pulp capping after MTA was introduced as a pulp-capping agent. Its excellent biocompatibility and ability to form reparative dentine made it a reliable pulp-capping material, but its long setting time and increased solubility are seen as drawbacks.

Recently, new calcium silicate materials have emerged in the market. Biodentine™ (Septodont, Saint-Maur-des-Fossés, France) is a modified MTA-like material designed to overcome the limitations of MTA when used as a pulp-capping material. It is a dentine substitute composed of tricalcium silicate cement, zirconium oxide, and calcium carbonate powder, which are enclosed in a capsule. The liquid in the ampule includes water, calcium chloride, and a water-based polymer [4]. TheraCal LC® (Bisco Inc., Schaumburg, IL) is a light-curable resin-modified calcium silicate-based material that can be used as a pulp-capping agent or a protective liner for restorative materials, cement, or other base materials in a single-paste format [4]. Bio-C Temp (Angelus, Brazil) is a ready-to-use bioceramic paste. Its primary ingredients are calcium silicates that, when hydrated, generate calcium hydroxide, which separates into Ca2+ and OH. The hydroxyl ions (OH-) that are discharged have a significant impact on the pH of the surrounding tissue, rendering it unsuitable for bacterial growth. It can be used in cases of deep caries, as it can induce calcific barrier formation [5]. The current study was conducted to evaluate the antimicrobial properties of these four calcium silicate-based pulp-capping agents.

## Materials and methods

The pulp-capping agents chosen for this study were divided into four groups: Group I (Dia-Root™ Bio MTA), Group II (TheraCal LC®), Group III (Bio-C® Temp), and Group IV (Biodentine™). The ATCC strain (29212) of *E. faecalis* and the ATCC strain (25175) of *S. mutans*, obtained from "HiMedia," were used. Both organisms were cultured in brain heart infusion broth at 37° for 24 hours. The purity of each culture was confirmed with gram staining, and the morphology was confirmed through microscopic evaluation. The density of the bacterial suspension was standardized to a concentration of 1.5 × 108 and compared with 0.5 McFarland units of the barium sulfate standard. Nine blood agar plates and nine Mueller-Hinton agar (MHA) were used. The total sample size was 72, with nine per group at a 5% level of significance, 80% power, and an effect size of 0.5 using the software G*Power (version 3.1; Heinrich Heine University Düsseldorf, Düsseldorf, Germany).

Agar diffusion test

**Figure 1:**
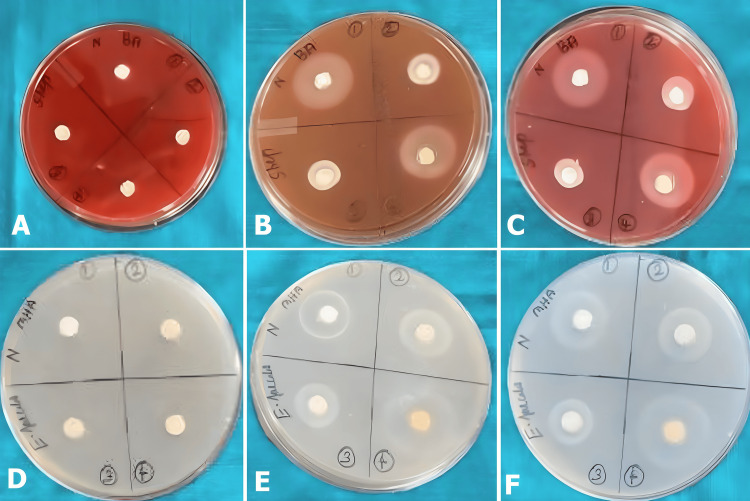
Zone of inhibition formed by antibacterial activity of pulp-capping agents (A) The prepared wells were filled with pulp-capping agents in a blood agar plate with *Streptococcus ​​​mutans*. (B) Zones of inhibition for *Streptococcus* *mutans* at 24 hours. (C) Zones of inhibition for *Streptococcus mutans* at 48 hours. (D) Prepared wells were filled with pulp-capping agents in an MHA plate with *Enterococcus faecalis*. (E) Zones of inhibition for *Enterococcus faecalis* at 24 hours. (F) Zones of inhibition for *Enterococcus faecalis* at 48 hours. MHA: Mueller-Hinton agar.

A microbiological assessment was conducted with agar diffusion test under aseptic conditions. Using blood agar, 0.5 ml of *S. mutans* suspension was transferred to nine petri dishes, while the *E. faecalis *suspension was transferred to the other nine petri dishes with MHA. Each of the petri dishes was divided into four sections, and four wells were prepared in each section. They were equidistant from each other and had a depth and diameter of 4 mm. This was immediately filled with freshly manipulated test materials. All the agar plates were incubated at 37°C, and the zone of inhibition was evaluated at 24 and 48 hours (Figure 1) by measuring the point where the growth ceased using a digital caliper. The readings were tabulated and subjected to statistical analysis.

## Results

The data were analyzed using IBM SPSS (Statistical Package for Social Sciences) software version 26 (IBM Corp., Armonk, NY). Descriptive statistics were used to summarize the data. The normality of the data was checked using the Shapiro-Wilk test. The mean values among the four groups were compared using a one-way analysis of variance (ANOVA) test. Tukey’s honest significant difference (HSD) post-hoc tests were also conducted for a detailed pairwise comparison. The level of significance was fixed at p ≤ 0.05; any value less than or equal to 0.05 was considered statistically significant.

Following a 24-hour incubation period, Group IV (Biodentine™) demonstrated the largest mean zone of inhibition (15.8 mm) against *S. mutans*. This was closely followed by Group I (Dia-Root™ Bio MTA), which measured 14.56 mm, and Group II (TheraCal LC®), which measured 12.56 mm. Group III (Bio-C ® Temp) had the lowest mean value of zone of inhibition (9.61 mm). After 48 hours, the greatest mean zone of inhibition was demonstrated by Group IV (16.44 mm), followed closely by Group I (15.22 mm), and Group II (13.11 mm). The lowest value was observed in Group III (10.11 mm; see Table 1).

**Table 1:** Descriptive statistics for Streptococcus mutans inhibition zones at 24 and 48 hours MTA: Mineral trioxide aggregate.

Time points	Group	N	Mean	Std. deviation	Std. error	95% Confidence interval for mean		Minimum	Maximum
						Lower bound	Upper bound		
24 hours	Dia-Root Bio MTA	9	14.56	0.88	0.29	13.88	15.23	13.00	16.00
	TheraCal LC	9	12.56	0.53	0.18	12.15	12.96	12.00	13.00
	Bio-C Temp	9	9.61	0.70	0.23	9.08	10.15	9.00	11.00
	Biodentine	9	15.83	0.79	0.26	15.23	16.44	15.00	17.00
48 hours	Dia-Root Bio MTA	9	15.22	0.79	0.26	14.61	15.83	14.00	16.00
	TheraCal LC	9	13.11	0.78	0.26	12.51	13.71	12.00	14.00
	Bio-C Temp	9	10.11	0.33	0.11	9.85	10.37	10.00	11.00
	Biodentine	9	16.44	0.88	0.29	15.77	17.12	15.00	18.00

Tukey’s HSD test revealed that there was a significant difference among the groups, as the p-value was less than 0.05 (Table 2).

**Table 2:** Post-hoc multiple comparison of groups based on Streptococcus mutans inhibition zones at different time points * The mean difference is significant at the 0.05 level. HSD: Honest significant difference.

Dependent variables		(I) Group	(J) Group	Mean difference (I-J)	Std. error	P-value	95% Confidence interval	
							Lower bound	Upper bound
24 hours	Tukey HSD	1.00	2.00	2.00^*^	0.35	0.000	1.06	2.94
			3.00	4.94^*^	0.35	0.000	4.00	5.88
			4.00	-1.28^*^	0.35	0.004	-2.22	-0.34
		2.00	1.00	-2.00^*^	0.35	0.000	-2.94	-1.06
			3.00	2.94^*^	0.35	0.000	2.00	3.88
			4.00	-3.28^*^	0.35	0.000	-4.22	-2.34
		3.00	1.00	-4.94^*^	0.35	0.000	-5.88	-4.00
			2.00	-2.94^*^	0.35	0.000	-3.88	-2.00
			4.00	-6.22^*^	0.35	0.000	-7.16	-5.28
		4.00	1.00	1.28^*^	0.35	0.004	0.34	2.22
			2.00	3.28^*^	0.35	0.000	2.34	4.22
			3.00	6.22^*^	0.35	0.000	5.28	7.16
			3.00	6.22^*^	0.35	0.000	5.25	7.20
48 hours	Tukey HSD	1.00	2.00	2.11^*^	0.34	0.000	1.18	3.04
			3.00	5.11^*^	0.34	0.000	4.18	6.04
			4.00	-1.22^*^	0.34	0.006	-2.15	-0.29
		2.00	1.00	-2.11^*^	0.34	0.000	-3.04	-1.18
			3.00	3.00^*^	0.34	0.000	2.07	3.93
			4.00	-3.33^*^	0.34	0.000	-4.27	-2.40
		3.00	1.00	-5.11^*^	0.34	0.000	-6.04	-4.18
			2.00	-3.00^*^	0.34	0.000	-3.93	-2.07
			4.00	-6.33^*^	0.34	0.000	-7.27	-5.40
		4.00	1.00	1.22^*^	0.34	0.006	0.29	2.15
			2.00	3.33^*^	0.34	0.000	2.40	4.27
			3.00	6.33^*^	0.34	0.000	5.40	7.27

**Figure 2:**
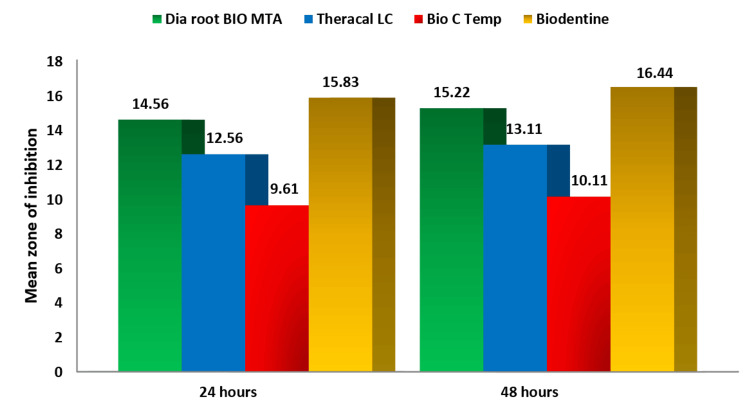
Comparison of the mean zones of inhibition at 24 and 48 hours among the study groups for Streptococcus mutans MTA: Mineral trioxide aggregate.

Group IV showed the highest mean value of zone of inhibition at 24 and 48 hours against *S. mutans* (Figure 2).

After analyzing the data descriptively at both 24 and 48 hours, it was clear that Group IV had the highest average inhibition zone against *E. faecalis*, with measurements of 20.56 mm at 24 hours and 21.28 mm at 48 hours. Groups I and II showed inhibition zones of 20.06 and 18.22 mm, respectively, at 24 hours, and 20.56 and 18.67 mm, respectively, at 48 hours. In contrast, Group III displayed the lowest values: 14.11 mm at 24 hours and 15.22 mm at 48 hours (Table 3).

**Table 3:** Descriptive statistics for Enterococcus faecalis inhibition zones at 24 and 48 hours MTA: Mineral trioxide aggregate.

Time points	Group	N	Mean	Std. deviation	Std. error	95% Confidence interval for mean		Minimum	Maximum
						Lower bound	Upper bound		
24 hours	Dia-Root Bio MTA	9	20.06	1.33	0.44	19.03	21.08	18.00	22.00
	TheraCal LC	9	18.22	0.97	0.32	17.48	18.97	17.00	20.00
	Bio-C Temp	9	14.11	0.78	0.26	13.51	14.71	13.00	15.00
	Biodentine	9	20.56	0.73	0.24	20.00	21.11	19.00	21.00
48 hours	Dia-Root BIOMTA	9	20.56	1.13	0.38	19.69	21.42	19.00	22.00
	TheraCal LC	9	18.67	0.87	0.29	18.00	19.33	18.00	20.00
	Bio-C Temp	9	15.22	0.44	0.15	14.88	15.56	15.00	16.00
	Biodentine	9	21.28	0.67	0.22	20.77	21.79	20.00	22.00

**Figure 3:**
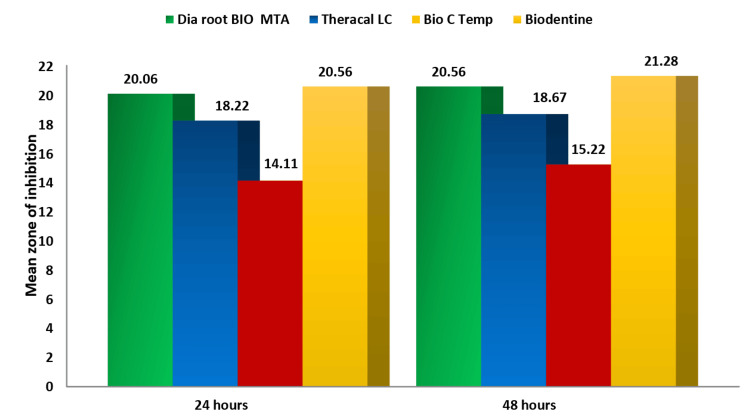
Comparison of mean zones of inhibition at 24 and 48 hours among the study groups for Enterococcus faecalis MTA: Mineral trioxide aggregate.

Multiple comparisons among each group were done using Tukey’s HSD test, which suggested no significant difference between the antibacterial efficacy of Groups I and IV (Table 4). However, Group IV had the highest mean value of zone of inhibition at both 24 and 48 hours (Figure 3).

**Table 4:** Post-hoc multiple comparison of groups based on Enterococcus faecalis inhibition zones at 24 and 48 hours * The mean difference is significant at the 0.05 level. HSD: Honest significant difference.

Multiple comparisons								
Dependent variables		(I) Group	(J) Group	Mean difference (I-J)	Std. error	P-value	95% Confidence interval	
							Lower bound	Upper bound
24 hours	Tukey HSD	1.00	2.00	1.83^*^	0.46	0.002	0.58	3.09
			3.00	5.94^*^	0.46	0.000	4.69	7.20
			4.00	-0.50	0.46	0.704	-1.75	0.75
		2.00	1.00	-1.83^*^	0.46	0.002	-3.09	-0.58
			3.00	4.11^*^	0.46	0.000	2.86	5.37
			4.00	-2.33^*^	0.46	0.000	-3.59	-1.08
		3.00	1.00	-5.94^*^	0.46	0.000	-7.20	-4.69
			2.00	-4.11^*^	0.46	0.000	-5.37	-2.86
			4.00	-6.44^*^	0.46	0.000	-7.70	-5.19
		4.00	1.00	0.50	0.46	0.704	-0.75	1.75
			2.00	2.33^*^	0.46	0.000	1.08	3.59
			3.00	6.44^*^	0.46	0.000	5.19	7.70
48 hours	Tukey HSD	1.00	2.00	1.89^*^	0.38	0.000	0.85	2.93
			3.00	5.33^*^	0.38	0.000	4.29	6.38
			4.00	-0.72	0.38	0.258	-1.77	0.32
		2.00	1.00	-1.89^*^	0.38	0.000	-2.93	-0.85
			3.00	3.44^*^	0.38	0.000	2.40	4.49
			4.00	-2.61^*^	0.38	0.000	-3.65	-1.57
		3.00	1.00	-5.33^*^	0.38	0.000	-6.38	-4.29
			2.00	-3.44^*^	0.38	0.000	-4.49	-2.40
			4.00	-6.06^*^	0.38	0.000	-7.10	-5.01
		4.00	1.00	0.72	0.38	0.258	-0.32	1.77
			2.00	2.61^*^	0.38	0.000	1.57	3.65
			3.00	6.06^*^	0.38	0.000	5.01	7.10

## Discussion

The development of dental caries is a multifaceted process that begins with the formation of a structured biofilm that contains various cariogenic microbial communities [6,7]. *S. mutans* is recognized as one of the primary microorganisms responsible for causing dental caries [6,8]. *S. mutans* can induce low oral pH and thus contribute to the demineralization of tooth enamel by metabolizing carbohydrates [9]. *E. faecalis* is a gram-positive facultative anaerobe that can survive in hostile conditions, making disinfection difficult. Moreover, *E. faecalis* can withstand extreme conditions; thus, total eradication is difficult [10]. The presence of this bacteria can lead to the failure of pulp therapy. Radman et al. found that *E. faecalis* is the most predominant bacteria in deep carious lesions [11]. Partial or incomplete removal of caries can lead to pulp therapy failure.

Pulp-capping agents are biocompatible materials placed directly or indirectly over the pulp tissue, inducing the formation of a calcific barrier and thus preventing the progression of microorganisms to the root canal space and the periapical region. Pulp-capping materials should have good biocompatibility with the contacted dental tissue, including pulp, and should not cause inflammation, irritation, allergy, or toxic reactions [12]. These materials should also possess antibacterial properties and inhibit the progress of residual caries.

For many years, calcium hydroxide was the material of choice for direct pulp capping. Calcium hydroxide inhibits the growth of microorganisms and encourages the healing and defense mechanisms of pulp tissue. It also exerts direct antibacterial action due to its highly alkaline pH. One of the main drawbacks is that it cannot achieve a good seal to the tooth due to its high solubility and poor adherence to hard tissues [3].

To overcome these limitations, newer materials with better properties have been developed, including Dia-Root™ Bio MTA (Diadent Europe B.V., Almere, Netherlands), TheraCal LC (Bisco Inc., Schaumburg, IL), Bio-C® Temp (Angelus, Brazil), and Biodentine™ (Septodont, Saint-Maur-des-Fossés, France). Of these materials, Dia-Root™ Bio MTA, TheraCal LC, and Bio-C® Temp are the most recent, and little research is available on the antibacterial efficacy of these materials.

MTA is a widely accepted pulp-capping material due to its potential to promote wound healing of the dentin-pulp complex, making it a viable alternative to calcium hydroxide. MTA is primarily composed of tricalcium silicate, dicalcium silicate, and tricalcium aluminate, with bismuth oxide added for radiopacity. Its advantages include increased working time and the ability to function in moist environments. The material has excellent biocompatibility when applied to a pulp wound, superior sealing ability, low solubility, and the ability to inhibit bacterial invasion and induce dentin bridge formation. The calcium ions released from MTA have antibacterial effects and promote mineralization beneath the pulp exposure area, which helps maintain pulp vitality [13]. In addition, MTA prevents bacterial leakage if secured with a properly sealed restoration, promoting healing and protecting the pulp tissue.

However, conventional MTA has been documented to have various limitations when used in clinical settings. These limitations include its extended setting time, high cost, susceptibility to moisture interference, and limited compressive strength [13]. A new silicate-based variety, Dia-Root™ Bio MTA, has been introduced to address these limitations. Dia-Root™ Bio MTA is an advanced calcium silicate-based material designed to be easily manipulated and enhance the formation of calcific barrier with improved sealing capabilities [14].

Biodentine™ is a bioactive material comprising tricalcium silicate, dicalcium silicate, calcium carbonate, iron oxide, and zirconium oxide. It has many superior mechanical properties and a faster setting time compared to MTA [12]. In addition, the release of calcium ions has been observed to occur at a faster rate compared to other materials of a similar nature, making it a preferred choice for pulp-capping applications. Biodentine™ showcases the highest concentration of surface calcium among its counterparts [15]. Clinical evidence demonstrates the formation of dentine bridges when Biodentine™ is utilized for direct pulp capping in a range of studies [16]. Furthermore, Koruyucu et al. found that Biodentine™ has a similar antibacterial activity to MTA against *E. faecalis* [17].

TheraCal LC is a revolutionary calcium silicate resin-based substance that serves as an effective pulp-capping agent and a protective liner for dental restorations. This innovative material belongs to the category of light-curable MTA cement, marking it as a fourth-generation calcium silicate product. The release of calcium ions can trigger the formation of new mineralized hard tissues. Studies show that the amount of calcium ions released by TheraCal LC may stimulate the dental pulp and odontoblasts. Moreover, the material exhibits exceptional bonding capabilities with both teeth and restorative materials. The presence of hydroxyl ions (OH-) in TheraCal LC contributes to its impressive antibacterial properties. In addition, the material increases pH levels, which can lead to mild irritation of the pulp tissue and subsequent mineralization, facilitating optimal healing processes [4].

Bio-C® Temp is a calcium silicate-based material that produces calcium hydroxide that dissolves into Ca2+ and OH- ions on hydration. The released hydroxyl ions lead to increased pH, causing the environment to become inappropriate for bacterial growth. Although it is not a commonly used pulp-capping agent, it can be considered a pulp-capping material due to its composition and ability to alter the pH. A significant benefit of this product compared to calcium hydroxide pastes is its low solubility, which enables prolonged contact with the cavity. The material’s ability to form a calcific barrier also makes it suitable for use as a pulp-capping agent [18].

This study assessed the effectiveness of four calcium silicate-based materials as potential pulp-capping agents and their antibacterial properties. The results revealed that, of the four materials tested, Biodentine™ demonstrated the highest antibacterial efficacy against both *S. mutans* and *E. faecalis*, followed by Dia-Root™ Bio MTA and TheraCal LC. Bio-C® Temp showed the lowest inhibition zone value. It should be noted that Bio-C® Temp has different formulation and setting properties compared to the other pulp-capping materials; however, this study evaluated its potential as a pulp-capping material.

There was a statistically significant difference in the zone of inhibition between all four groups against *S. mutans.* Biodentine™ and Dia-Root™ Bio MTA were highly effective against* E. faecalis* bacteria, with no significant difference between the two materials. TheraCal LC also showed antibacterial properties, but its zone of inhibition was significantly lower than those of Biodentine™ and Dia-Root™ Bio MTA. Bio-C® Temp had the lowest mean zone of inhibition against both organisms at both time intervals.

Many clinical studies have tested the efficacy of different pulp-capping agents. This is relevant because a successful pulp-capping result can only be achieved if the pulp-capping material shows superior antibacterial properties. Many studies have found that Biodentine™ exhibits superior antibacterial activity compared to other pulp-capping materials [12,18]. Bhat et al. conducted a study on the antibacterial efficiency of different pulp-capping materials against *E. faecalis* and *S. mutans *using MTA and Biodentine™ and found that both materials showed high antibacterial efficacy. However, Biodentine™ produced a higher inhibition zone than MTA [12]. Although the difference was insignificant, Biodentine™ showed a greater impact, as in the current study. Similarly, Jain et al. [18] evaluated the antibacterial efficacy of Biodentine™ and MTA on *S. mutans* and *E. faecalis *and derived results similar to the current study. They concluded that both Biodentine™ and MTA showed high antibacterial efficacy, but a higher zone of inhibition was found in the Biodentine™ group [18].

In alignment with this study, Esteki et al. discovered that Biodentine™ demonstrated stronger antimicrobial effects against *E. faecalis* and *S. mutans *compared to MTA and the other materials evaluated in their research [19]. Hondares et al. found in their study that Biodentine™ exhibited the most significant growth inhibition effects against both *S. mutans* and *E. faecalis* when compared to MTA and other tested materials [20]. Vakil et al. conducted a study that demonstrated Biodentine's promising efficacy against *E. faecalis* and *S. mutans*, surpassing MTA in forming larger inhibition zones [21].

Bakır et al. conducted a comparative analysis of the antibacterial properties of various pulp-capping materials where their findings indicated that MTA demonstrated the highest antibacterial effectiveness, closely followed by TheraCal LC in their action against *S. mutans* and *E. faecalis*. In contrast, Biodentine™ exhibited considerably lower antibacterial performance than both MTA and TheraCal LC, which contradicts the results of this study [22].

Based on the result of the current study, Biodentine™ may be considered the preferred material for pulp capping over MTA, TheraCal LC, and Bio-C® Temp due to its superior antibacterial efficacy. In addition, Biodentine™ is an effective antibacterial agent against both *S. mutans *and *E. faecalis*, regardless of the duration of application. However, further clinical studies are necessary to validate these results.

This study’s limitations include that, as an in vitro study, it may not accurately reflect the conditions in the oral cavity. While the agar diffusion method is valuable in assessing the antibacterial properties of pulp-capping agents, it may not provide a complete picture of the antibacterial properties of the test materials. For example, some agents may have limited diffusion capabilities in agar, which can lead to underestimated results.

## Conclusions

All the tested pulp-capping agents showed varying degrees of antibacterial activity. However, Biodentine™ showed the highest antibacterial efficacy on both *S. mutans *and *E. faecalis*, followed by Dia-Root™ Bio MTA, TheraCal LC, and Bio-C® Temp. Biodentine™ can thus be recommended as an effective pulp-capping agent in the management of deep carious lesions. Further clinical studies are warranted to substantiate this claim.
